# Soft Drinks Containing Poisons

**Published:** 1911-07

**Authors:** Lyman F. Kebler

**Affiliations:** Chief Division of Drugs, U. S. Department of Agriculture


### Soft Drinks Containing Poisons.

BY LYMAN F. KEBLER, M. D., PH. D.,
Chief Division of Drugs, U. S. Department of Agriculture.

During the past decade soda-fountain specialties containing caffeine, extract of Kola nut and extract of coca leaf, the active principle of which is cocaine, have been offered in considerable quantities and, due to extensive and attractive advertising, both as beverages and as headache remedies and nerve tonics, their sale has reached large proportions.

The first appearance of preparations of this type was in the South in the 80's, their introduction following the success which Moxie had attained in the East, though this particular drink was of entirely different character. From the South the demand spread to other sections, and the number of products has increased until at the present time there are probably over one hundred of them bottled and sold all over the United States. The greatest demand is still in the South, however, and almost every drug store, confectionery shop and fruit stand has its favorite product on sale. The carbonated goods in bottled form are offered on the trains. People of all classes, young and old, delicate women and even little children consume these beverages indiscriminately and no warning is ever given of the baneful effect of the powerful habit-forming drugs concealed therein. It is therefore small wonder that the prevalence of the so-called "coca-cola fiend" is becoming a matter of great importance and concern.

It is well known that some of these products are mixed under the most unsanitary conditions. The sugar, water, and drug material will be dumped into a pot standing in the cellar of some low building, or even a stable, where the ceiling is covered with dust, cobwebs, and dirt of all descriptions and the floor littered with filth. The steam from boiling kettle, condensing on the ceiling, collects the dirt in the drops of water and this falls back into the mixture. Again the syrup boils over on the floor and a sticky mass remains which soon collects straw and filth of all descriptions and becomes a rendezvous for flies and other vermin, for usually no attempt is made to clean up.

Judging from the names of most of these products it would appear that extract of Kola nut is one of the chief ingredients, and while in certain instances the drug is undoubtedly present, in most cases the caffeine has been added as the alkaloid caffeine obtained from refuse tea sweepings or made artificially from uric acid occurring in the guano deposits of South America, or in the citrated form and the syrup colored with caramel. The cocaine found is usually added in the form of extract of coca leaf. Some of the manufacturers claim that the extract used is prepared from a decocainized coca leaf, the refuse product discarded in the manufacture of cocaine.—President Roosevelt's *Homes Commission Report* (1909).—*Scientific Temperance Journal,* Boston.

---

In pyuria of prostatic origin—as shown by massage—if there has been no recent infection and, especially, if the pus is germ-free, prostatic calculi should be thought of. A skiagraph will determine their presence or absence.—*American Journal of Surgery.*

